# Diffuse and disseminated cutaneous leishmaniasis: clinical cases experienced in Ecuador and a brief review

**DOI:** 10.1186/s41182-016-0002-0

**Published:** 2016-03-14

**Authors:** Yoshihisa Hashiguchi, Eduardo L. Gomez, Hirotomo Kato, Luiggi R. Martini, Lenin N. Velez, Hiroshi Uezato

**Affiliations:** Facultad de Medicina, Universidad Catolica de Santiago de Guayaquil, Guayaquil, Ecuador; Proyecto Prometeo, Secretaría Nacional de Educación Superior, Ciencia, Tecnología e Innovación (SENESCYT), Quito, Ecuador; Department of Parasitology, Kochi Medical School, Kochi University, Kochi, Japan; Departamento de Medicina Tropical, Facultad de Medicina, Universidad Católica de Santiago de Guayaquil, Guayaquil, Ecuador; Servicio Nacional de Erradicacion de la Malaria (SNEM), Ministerio de Salud, Guayaquil, Ecuador; Laboratory of Parasitology, Department of Disease Control, Graduate School of Veterinary Medicine, Hokkaido University, Sapporo, Japan; Departamento de Parasitología, Instituto Nacional de Investigación de la Salud Pública, Guayaquil, Ecuador; Department of Dermatology, Faculty of Medicine, University of the Ryukyus, Okinawa, Japan

**Keywords:** Cutaneous leishmaniasis, Diffuse, Disseminated, Differentiation, Ecuador, Old and New Worlds

## Abstract

**Background:**

In Ecuador, cutaneous leishmaniasis (CL) is prevalent countrywide, but only one case of diffuse-CL and two cases of disseminated-CL were experienced during our research activities more than 30 years from 1982 to date. These three patients suffered from multiple lesions distributed at a wide range of the body surface, revealing difficulty to clinically differentiate each other.

**Methods:**

There is a considerable confusion of the use and/or differentiation of the terminologies (terms) between the two disease forms, diffuse-CL and disseminated-CL. One of the aims of the present study is to clarify the difference between the two disease forms, mainly based on the cases experienced in Ecuador.

**Results:**

The disseminated-CL case newly reported here was clinically very similar to the diffuse-CL case, but the former showed the following marked differences from the latter: (1) the organisms isolated were identified as the parasites of *Leishmania* (*Viannia*) *guyanensis*/*panamensis*, which are also known as the causative agents of disseminated-CL in different endemic countries of the New World; (2) the patient was sensitive against antimonials; and (3) mucosal involvement was observed, which is never observed in diffuse-CL.

**Conclusions:**

In the text, three clinical cases, one diffuse-CL and two disseminated-CL, were presented. Furthermore, a bibliographic comparison of the features between the two disease forms was made, and a brief comment was also given.

## Background

Leishmaniases are one of the vector-borne diseases, caused by obligate intra-macrophage protozoan parasites of the genus *Leishmania*, with subgenera *Leishmania* and *Viannia*. The disease is transmitted by the bite of infected female phlebotomine sand flies of the genera *Phlebotomus* and *Lutzomyia* in the Old and New Worlds, respectively. In humans, about 20 *Leishmania* species are responsible for a wide spectrum of clinical manifestations, ranging from localized cutaneous leishmaniasis (CL) to those producing diffuse-CL and disseminated-CL, mucocutaneous (MCL), and visceral (VL) forms known as kala-azar. These different types of the disease are endemic in many tropical and subtropical regions and responsible for increasing health problems in large parts of the world [1, 2].

The diffuse-CL case was reported for the first time by Convit and Lapenta from Venezuela in the New World and then by Destombes et al. from Ethiopia in the Old World [3, 4]. The former authors reported one case as an atypical clinical form in Spanish version entitled “*Sobre un caso de leishmaniasis tegmentaria de forma diseminada*” (one case of disseminated form of tegumentary leishmaniasis) in a Venezuelan medical journal. Later, the latter authors reported a similar case from Ethiopia in French version entitled “*Leishmaniose cutanee nodulaire disseminee en Ethiopie*” (disseminated cutaneous nodular leishmaniasis in Ethiopia). The authors of the two articles used the same terminology “disseminate” against diffuse-CL forms in the title. After these reports, some workers like to use “disseminate” against diffuse-CL and others used “diffuse” against the same disease form (diffuse-CL). On the other hand, disseminated-CL cases in the New World were reported for the first time by Costa et al. and then by Carvalho et al. and Turetz et al. from Brazil [5–7]. The disseminated-CL is mainly found in the New World.

Thus, for many years, diffuse-CL and disseminated-CL remain clinical forms of the well-classified skin manifestations of the disease. Still, however, there is a considerable confusion of the utilization or differentiation of the terminology between the two disease forms. We therefore tried to emphasize the correct and precise differentiation of the two clinical forms. In the text, three clinical cases, one diffuse-CL and two disseminated-CL, were presented. Furthermore, a brief review was given, performing a bibliographic comparison between the two disease forms. Informed consent was obtained from the subjects who participated.

## Methods

### Patient’s origin and identification of *Leishmania* species

#### Patients’ localities

**Fig. 1:**
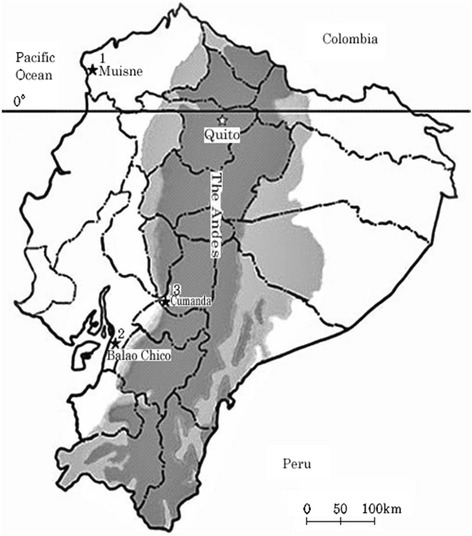
Map of Ecuador, showing the localities from which patients with diffuse-CL and disseminated-CL cases were reported. *1* Muisne, Esmeraldas province: one case of diffuse-CL; *2* Balao Chico, Guayas province: one case of disseminated-CL; *3* Cumanda, Chimborazo province: one case of disseminated-CL

We experienced one case of diffuse-CL from Muisne, Esmeraldas province, and two cases of disseminated-CL from Balao Chico, Guayas province, and more recently from Cumanda, Chimborazo province, respectively (Fig. 1).

#### Clinical samples from patients

Clinical samples from diffuse-CL (Muisne) and disseminated-CL (Cumanda) were taken by scraping the margin of active lesions of the patients, spotted onto a FTA classic card (Whatman, Newton Center, MA), and stored at room temperature. Disks 2 mm in diameter were punched out from each filter paper and washed three times with FTA purification reagent (Whatman) and once with Tris-EDTA buffer. The disks were air-dried and directly subjected to PCR amplification. Nested PCR was performed to amplify the *Leishmania* cytochrome b (*cyt* b) gene from a patient specimen collected on the FTA cards. Primers used for the nested PCR were L.cyt-AS (5′-GCGGAGAGRARGAAAAGGC-3′) and L.cyt-AR (5′-CCACTCATAAATA TACTATA-3′) for the first PCR and L.cyt-S and L.cyt-R for the second PCR [8, 9].

#### Montenegro (Leishmanin) skin test and recombinant kinesin-like antigen (rK39) kit test

To see the cellular immune response of the subjects examined, intradermal skin test antigen derived from *Leishmania* (*Viannia*) *panamensis* promastigotes was applied to the subjects; more than 5 mm in diameter of the induration was considered as positive [10]. Besides, recombinant kinesin-like K39 kit test (rK39 antigen of *L.* (*Leishmania*) *chagasi* produced by InBios International (Kalazar Detect™ Rapid Test kit, Seattle, WA, USA )) was applied to the sera of the subjects, in order to see humoral immune response.

#### Phylogenetic analysis

Cytochrome b (*cyt* b) gene sequence was performed with CLUSTAL W software [11] and examined using the program MEGA (Molecular Evolutionary Genetics Analysis) version 5.1 [12]. Phylogenetic trees were constructed by the neighbor-joining method with the distance algorithms available in the MEGA package. Bootstrap values were determined with 1000 replicates of the data sets. The database for phylogenetic analyses consisted of *cyt* b gene sequences from GenBanks of 12 *Leishmania* species.

## Results

### Diffuse and disseminated cutaneous leishmaniasis in Ecuador

#### A case of diffuse cutaneous leishmaniasis (diffuse-CL)

**Fig. 2:**
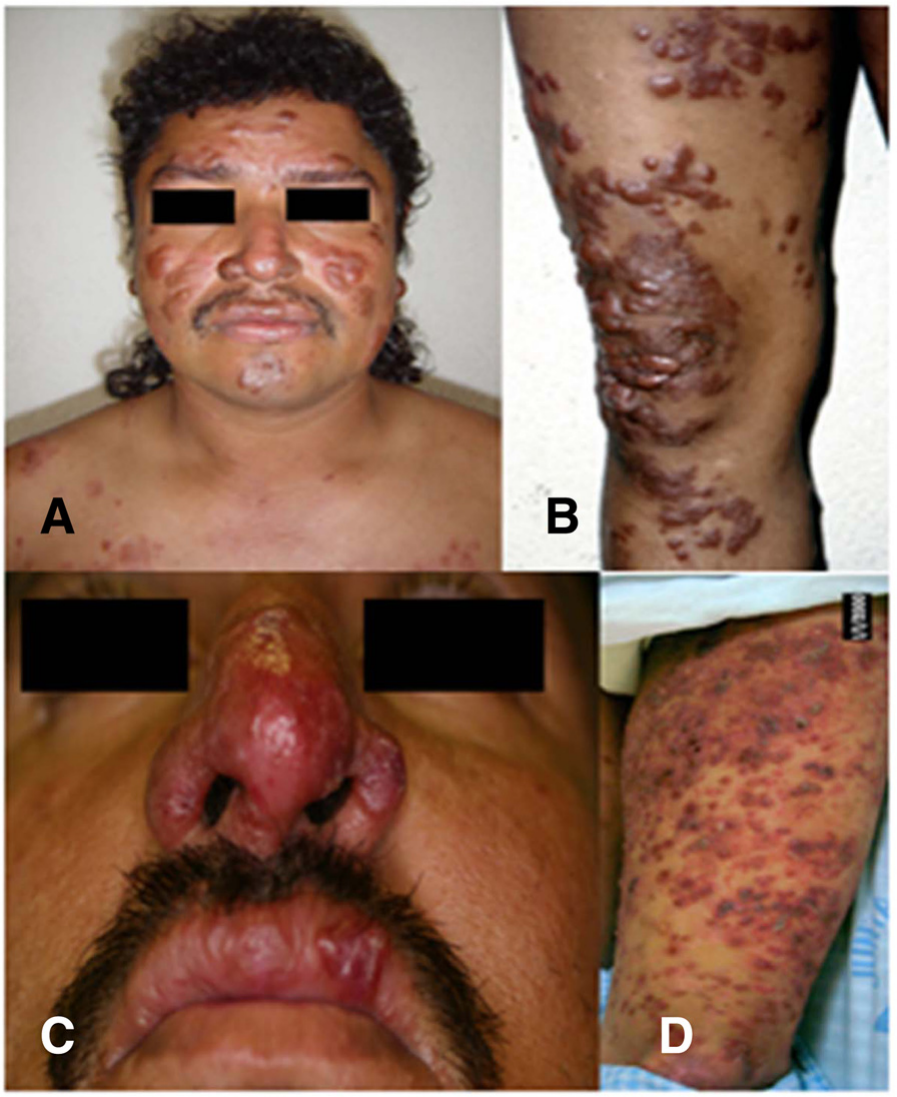
**a**, **b** Diffuse-CL case: **a** Rice-grain to thumb-sized reddish nodules and papules were observed on the ear lobes, auricles, face, and shoulders. **b** Various types of eruptions, such as papules, nodules, infiltrated erythemas, and brownish-colored freckles, were found on the lower extremities. Induration of the lesions was palpable. **c**, **d** Disseminated-CL case: **c** Superficial involvement of mucosae was recognized on the nose and lip, with partial ulceration. **d** Multiple and confluent lesions were observed on a wide range of the lower extremities with partial ulceration

We experienced a case of diffuse-CL [13]. The patient was a 24-year-old male (Fig. 2a, b) and was born and grew up in a small village near the Pacific coast of Ecuador, San Ignacio, Muisne, Esmeraldas province, a rural and mountainous area, located at 20 m above sea level (a.s.l.) (Fig. 1 (1)). In our epidemiological survey in the area, none of the patient’s family members and neighboring people revealed diffuse-CL cases (Hashiguchi et al., unpublished data). When the patient was 16 years old, papules appeared on his left knee and right cheek. The eruption gradually increased in size and number. For about 2 years, he received various medications without confirmed diagnosis. Then, he was diagnosed clinically with leprosy and received the specific medication (multidrug treatment: dapsone, rifampicin, and clofazimine) for 1 month. After precise clinical and laboratory examinations, he was finally diagnosed with a diffuse-CL at the Department of Parasitology, National Institute of Tropical Medicine and Hygiene, Guayaquil city, Ecuador, with positive smears for *Leishmania* parasites. Montenegro (Leishmanin) skin test was negative, but tuberculin, candidine, and trichophytin skin tests revealed positive reactions, demonstrating the existence of specific anergy against *Leishmania* antigen. On the other hand, the rK39 kit test revealed a strong positive reaction, suggesting the high-level function of humoral immune system of the diffuse-CL patient.

**Fig. 3:**
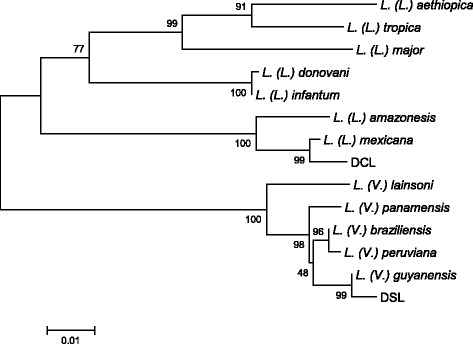
Phylogenetic tree of *cyt* b gene sequences among species. *Cyt* b genes of the *Leishmania* parasites were amplified and sequenced from patients with diffuse-CL (DCL) and disseminated-CL (DSL). A phylogenetic analysis of *cyt* b gene sequences was performed by the neighbor-joining method together with sequences from 12 *Leishmania* species. The *scale bar* represents 0.01 % divergence. *Bootstrap values* are shown *above* or *below branches*

Physical examinations revealed pea- to rice-grain-sized reddish papules on both the ear lobes and the auricles. Induration of the lesions was palpable. Miliary to pea-sized reddish papules were observed on the face. The papules and nodules were also observed on the left shoulder. Many miliary to hen-egg-sized papule, nodule, erythema, infiltrated erythema, and brownish-colored freckles were observed on the upper extremities. The surfaces of some of the nodules were scaly and crusted. Rice-grain to thumb-sized reddish papules grouped on the brownish-colored freckle lesion on the lower back were observed. Miliary-sized papules were scattered showing satellite lesions around the plaque-like lesions. Thumb- to palm-sized erythema was observed on the thigh. No loss of sensation was recognized. Various types of eruptions, such as papules, nodules, infiltrated erythema, and brownish-colored freckles, were observed on the lower extremities. There were no eruptions on the hands, the feet, the scalp, and the axillary, epigastric, inguinal, perineal, and anal regions, except a few verruca vulgaris lesions on the dorsal aspect of the left hand. There was no sensory loss on all over the body surface and no hypertrophy of the peripheral nerve. Numerous *Leishmania* amastigotes were observed in the stained smears from the skin lesion, though no acid-fast bacilli were observed in the slit-skin smear materials with Ziehl-Neelsen staining. The parasite was identified as *L.* (*L.*) *mexicana* by PCR analysis (Fig. 3).

Histological findings of the specimen taken from nodules on the thigh showed hyperkeratosis and mild acanthosis of the dermis. There was dense cellular infiltration in the dermis. Proliferations of blood vessels were also visible. In the foamy cells, innumerable *Leishmania* amastigotes were observed. No epithelioid cell granuloma was observed as seen in leprosy. No acid-fast bacilli were observed in the dermis. The patient was treated with meglumine antimoniate (Glucantime®) at a dose of 10 mg/kg/day and sodium stibogluconate (Pentostam®) at a dose of 20 mg/kg/day for 28 days with each drug; partial resolution of skin lesion was observed but relapsed soon after discontinuation of the treatment. In these treatments, the density of parasite on smear specimens was significantly reduced immediately after the beginning of each treatment but never disappeared completely. Thus, relapses were followed by 5- or 6-month intervals after each of the slight clinical improvements. Therefore, the patient had to receive repeatedly the intramuscular injection of Glucantime® after each relapse. Following this antimonial treatment, the patient received also pentamidine (3–4 mg/kg intramuscularly once or twice for a week) but had to be discontinued because of severe adverse reactions such as hypotension, gastrointestinal symptoms, and syncope, without revealing a marked clinical and parasitological healing. Subsequently, the patient was treated with oral itraconazole (200 mg/day for 3 months), mefloquine (4.2 mg/kg/day for 6 days, repeated with a 3-week interval), and artesunate (6.7 mg/kg/day for 3 days, repeated with a 2-week interval), but compete healing was not obtained by any of these drugs. These mixed and alternative treatments were performed one after another because of the lack of any effective drugs for this diffuse-CL patient. The patient was also treated with oral miltefosine [13, 14]. The drug (Impavido®) was started at a dose of 2.5 mg/kg/day and continued for 5 months. However, 2 months after discontinuing miltefosine, small papular lesions appeared and the slit-skin smears and aspirates of lesions had become positive for *Leishmania* parasites. Miltefosine was re-started at the same dose, but no clinical or parasitological response was observed even after 2 months of re-treatment, and skin lesions continued to grow and spread over the entire body [14]. No specific and efficient drug is available for the diffuse-CL in the world at the moment.

### Two cases of disseminated cutaneous leishmaniasis (disseminated-CL)

#### The first case of disseminated-CL

We diagnosed the first case of disseminated-CL in Ecuador and named it as “generalized CL” [15]. The patient was a 40-year-old female, as of the time of examination (August 1979). The patient came from a small village, Balao Chico, Guayas province, a mountainous area located at 200 m a.s.l. (Fig. 1 (2)). At the early phase of the disease, the lesion appeared on the lumbar region, and then papular lesions were seen, which became pustulous vesicles at the right lumber region and disseminated all over the body, demonstrating 308 generalized lesions counted. The patient revealed a marked right lumbar pain, and some of the lesions in the body surface suspecting herpes zoster were observed. The patient was admitted for 16 days in a private hospital and then was taken to a national hospital “Hospital Luis Vernaza” in Guayaquil city, Ecuador. She was admitted to the hospital under the suspected diagnosis of smallpox, being registered No. 03739 in August 1979, without a precise diagnosis. As there was no improvement of her lesions on the 15th day of admission, she was taken to receive differential diagnosis at the National Institute of Tropical Medicine and Hygiene, Guayaquil city, Ecuador. The presumptive clinical diagnoses were paracoccidioidomycosis, cutaneous leishmaniasis, or staphylococcal infections. Firstly, the case was considered as paracoccidioidomycosis suggesting a generalized cutaneous form of the disease. But either *Paracoccidioides braziliensis* or *Staphylococcus aureus* were not observed in the examination of ulcer materials. Laboratory examinations revealed positive for betahemolytic streptococcus and sensitive for erythromycin, cephalexin, amikacin, and ampicillin and stool examination positive for *Trichuris trichiura*, *Ascaris lumbricoides*, and *Ancylostoma duodenale*.

Most of the ulcer was crusty. When lifting the crusts, they were excavated with a purulent fluid because of sever secondary infections. The ulcers had different sizes ranging from 0.5 to 20 mm in diameter. Some lesions had thick, prominent, and adhered crusts that bleed easily when they were separated. Others were small crusty ulcers that left an excavation, and some of them were erythematous and papulous, and confluent plaques were found on the lumbar-dorsal region. Several smear samples from diverse regions of the body surface were taken at random. The samples were positive for the *Leishmania* amastigote, though the number was scanty in smear specimens. The parasite isolated was identified as *L.* (*V.*) *panamensis* (designated as MHOM/EC/95/NA-03) by ELISA species-specific monoclonal antibodies (serodeme) analysis [16]. No Montenegro skin test and rK39 kit test were performed in the case. The patient responded well to Glucantime® treatment (6.5 mg/kg/day for 12 days). In this case, both metronidazole per os and its ointment were also used as a supplementary treatment; probably, these accelerated the patient’s healing within a short regimen, in spite of a relatively low dose of the drug.

#### The second case of disseminated-CL

**Fig. 4:**
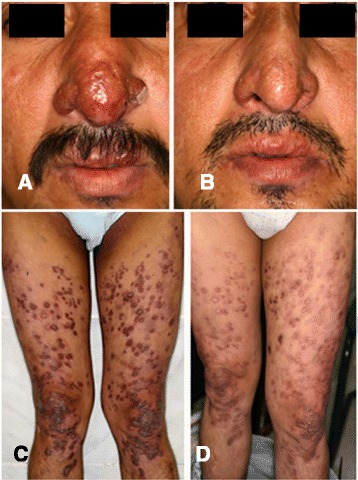
Disseminated-CL case, before and after the treatment. At the time of our examination, the patient already received injections of nine ampoules of trivalent antimonials (Repodral®) at the Cumanda health center. After finishing several courses of the treatment, he received another drug, pentavalent antimonials (Glucantime®) during about 5 months, responding well to the treatment. **a**, **c** Before the treatment, photographed on February 26, 2006. **b**, **d** After the treatment, photographed on August 17, 2006

More recently, we experienced the second case of disseminated-CL in Ecuador, which was clinically very similar to diffuse-CL. The patient was a 43-year-old male, as of February 22, 2006 (Fig. 2c, d), and came from a small community “Barrio 28 de Enero,” Chimborazo province, a subtropical mountainous area at the beginning of Andean slopes, located at 500 m a.s.l. (Fig. 1 (3)). The lesion began with an insect bite-like papular lesion on the forearm. Then, the lesions appeared on the right ear and the nose. About 2 years later, lesions appeared on a wide range of the lower extremities, followed by multiple and confluent lesions on the abdomen, thorax, body side, and thigh (photographs not shown). A superficial involvement of mucosae of the nose and upper lip was observed as seen in Fig. 4a. At the time of examination in the National Institute of Tropical Medicine and Hygiene, the patient already received injections of nine ampoules of trivalent antimonials Repodral® (20 mg/kg/day) at a rural health center (Cumanda, Guayas province). After finishing several courses of the treatment, the patient received Glucantime® (10 mg/kg/day) during about 5 months and responded well to the treatment. Montenegro skin test showed positive with a 17-mm induration, and rK39 kit test revealed a strongly positive band, suggesting a higher level of functions of the humoral immune system(s). Smear specimens, skin biopsies, syringe aspirates for in vitro cultures, and materials on FTA® classic cards (Whatman®) were taken from the active lesions. The smear specimen revealed positive for the *Leishmania* amastigote with a scanty number. Promastigotes grown in vitro had a tendency to decrease gradually after several days of cultivation and then totally disappeared, probably because of the effect of previous treatment with Repodral® before the material aspiration for culture. The parasites from the biopsy and FTA® card materials were identified as *L.* (*V.*) *guyanensis* by PCR analysis (Fig. 3). Thus, the case was clinically very similar to diffuse-CL but showed the following marked differences: (1) the organisms isolated were identified as the subgenus *Viannia*, *L.* (*V.*) *guyanensis*, which is also known as one of the causative agents of disseminated-CL in different endemic countries of the New World; (2) the patient was sensitive against antimonials; and (3) the superficial involvement of mucosae was recognized as seen in Fig. 2c, such a mucosal sign is never seen in diffuse-CL cases. The patient was alcoholic, and complications of his liver cirrhosis led to the unexpected death after healing of disseminated-CL.

## Discussion

In Ecuador, there are 24 provinces; among them, leishmaniases are prevalent in 21 provinces except Galapagos Is. As the causative agent of the *Leishmania* spp., there are eight species in total, *L.* (*L.*) *mexicana*, *L.* (*L.*) *amazonensis*, *L.* (*L.*) *major*-like, *L.* (*V.*) *braziliensis*, *L.* (*V.*) *guyanensis*, *L.* (*V.*) *panamensis*, *L.* (*V.*) *naiffi* [9, 17, 18], and the newly found *L.* (*V.*) *lainsoni* (Kato et al., unpublished data). In the country, localized CL is the most frequent clinical feature of the disease, followed by MCL cases mainly seen in the Ecuadorian Amazonian region [17, 18]. Only one diffuse-CL and two disseminated-CL cases in Ecuador were confirmed parasitologically and/or molecular biologically in our research activities from 1982 to date. No VL case was recorded, and its suspected vector species, *Lutzomyia longipalpis* and *L. evansi*, are not recorded among 29 species of man-biting sand flies [19]. The present diffuse-CL and disseminated-CL cases are found in the littoral northern (Esmeraldas) and southern (Guayas and Chimborazo) Ecuadorian regions. There will be a possibility of the presence of some unreported cases, especially in the Ecuadorian Amazonian regions, where the disease with diverse clinical manifestations are highly endemic, caused by different *Leishmania* species mentioned above.

Leishmaniases are caused by the parasites of the genus *Leishmania*, in which two subgenera *Leishmania* and *Viannia* exist. In the Old World, only the subgenus *Leishmania* is prevailing, while in the New World, both subgenera *Leishmania* and *Viannia* are circulating. The subgenus *Leishmania* parasites are causative agents of diffuse-CL in both the Old and New Worlds. The causative agents of diffuse-CL in the Old World are *L.* (*L.*) *aethiopica* and to a lesser extent *L.* (*L.*) *major* [20] and *L.* (*L.*) *amazonensis* and *L.* (*L.*) *mexicana* in the New World [21]. In the Old World, the clinical form of diffuse-CL is uncommon other than east Africa, Ethiopia, and Kenya [4, 22], but one each of cases masquerading as leprosy in the context of human immunodeficiency virus (HIV) co-infection was reported from Burkina Faso [23] and from Senegal [20]. On the other hand, in the New World, species of the subgenus *Leishmania*, such as *L.* (*L.*) *mexicana* and *L.* (*L.*) *amazonensis*, are responsible for the diffuse-CL in a wide range of South and Central American countries [21]. Among them, *L.* (*L*.) *mexicana* is mainly reported from the Central American countries and from a part of the northern regions of the South America [24]. No diffuse-CL cases caused by species of the subgenus *Viannia* are reported to date. The diffuse-CL lesions grow and proliferate as nodules and plaques which slowly but relentlessly spread to cover the entire body, with the exception of the scalp, axillae, inguinal fold, palms of the hands, and soles of the feet [25–28]. The diffuse-CL is a rare disease entity, and no more than 1–2 cases are diagnosed in all of Brazil each year [29].

The use of the terms, diffuse-CL and disseminated-CL, are frequently cited with a considerable confusion in literatures. In Brazil, many disseminated-CL cases were reported [5, 7, 13, 25, 26, 28]. Among them, a disseminated-CL case with 425 lesions caused by *L.* (*V.*) *guyanensis* was diagnosed both clinically and parasitologically [30]; in the text, however, the terminology (term) of both “disseminated” and “diffuse” against the disseminated-CL case was used without precisely differentiating the two disease forms, diffuse-CL and disseminated-CL. Nowadays, clear differences between the two forms were accumulated in literatures as shown in Table 1 [7, 15, 28, 30, 31].

**Table 1:** A bibliographic summary of the differences between diffuse-CL and disseminated-CL cutaneous leishmaniasis in the New and Old Worlds

Characteristics	DCL	DSL
Main agents		
In the New World	*L.* (*L.*) *amazonensis*	*L.* (*V.*) *braziliensis* group
	*L.* (*L.*) *mexicana*	*L.* (*L.*) *mexicana* group
In the Old World	*L.* (*L.*) *aethiopica*	No specific case reports other than HIV co-infection
	*L.* (*L.*) *major*	
No. of parasites in lesions/smears	Massive/abundant in smear (uncontrolled parasite growth)	Scanty/rare in smear
Leishmanin skin test	Negative/poor (specifically anergic to *Leishmania* antigen; lack of cell-mediated immunity)	Strongly positive
Reaction to TB, PPD, and other antigens	Yes	Yes
Antibody response	Elevated, rK39+^a^	Elevated, rK39+^a^
Ulceration of lesions	Never (non-necrotizing)	Frequent (necrotizing)
Coalescence to form plaques	Frequent	Rare
Type of lesions	Nodules, papules, plaques, macules, erythema	Papules, nodules, ulcers (mixture of small lesions)
Typical lesion	Lepromatous	Acneiform
Response to drug/therapy	Resistant/poor, relapse frequently	Good/poor
Infection/clinical course	Chronic, persist 20 years or more, for a life long	Not chronic
Analogous to lepromatous leprosy	Yes	No
Affected ages and sex	All ages and sexes	Young adults, male^b^
No. of lesions	Often innumerable, plaques on body surface	0–300^b^, 308^c^, 425^d^, 745^e^
Mucosal involvement	No	Frequent^b^
Disease entity	Rare, less than 0.1 % of total CL in Venezuela^f^	Less than 2 % of CL^b^

^a^Hashiguchi et al., unpublished data

^b^Turetz et al. [7]

^c^Lazo and Hashiguchi [15]

^d^Couppie et al. [30]

^e^Sousa et al. [31]

^f^Convit et al. [28]

The most marked differences between diffuse-CL and disseminated-CL are the presence of multiple non-ulcerative nodular lesions, a poor T cell response to *Leishmania* antigen, and a high number of phagocytosed *Leishmania* parasites within macrophages found in the former but not in the latter [6]. Characteristics of the present diffuse-CL patient corresponded to the previously reported cases, showing non-ulcerative lesions, negative skin test, and innumerable amastigotes in phagocytes. Silveira et al. proposed two pathogenicity extremes, the hypersensitivity pole by MCL and the hyposensitivity pole by anergic/diffuse-CL (ADCL), and they recommended the use of the term “borderline/disseminated-CL (BDCL)” for the disseminated form, caused by parasites of the subgenera *Leishmania* and *Viannia*, which might be regarded as intermediate between localized CL and the extreme pathogenicity poles MCL and ADCL [32]. Disseminated-CL is distributed widely in the New World and is characterized by cutaneous lesions, which may be accompanied by mucosal involvement and demonstrate a tendency toward chronicity and relapse as well as resistance to standard treatment regimens; parasites of the subgenus *Viannia*, *L.* (*V.*) *braziliensis* and *L.* (*V.*) *guyanensis*/*panamensis*, have been identified as the major etiological agent of this subset of infections [6, 7, 30, 33]. The disseminated-CL is also characterized by the presentation of a large number of acneiform and papular skin lesions, sometimes more than 700, at different anatomical regions such as the body surface, face, limbs, and trunks [7]. Most of the disseminated-CL lesions appear simultaneously with or secondary to one or several ulcerated lesions of localized CL. In an area of northeastern Brazil where *L.* (*V.*) *braziliensis* is endemic, patients with disseminated-CL presented 10–300 lesions that were a mixture of acneiform, papular, nodular, and ulcerated types; 12 (29 %) of 42 patients had mucosal involvement; and the patients with disseminated-CL had lower levels of interferon-γ and tumor necrosis factor-α production, compared with localized-CL patients [7]. An association between disseminated-CL and *L.* (*V.*) *braziliensis* strain polymorphisms was suggested that molecular genotyping may provide markers to identify the strains likely to cause an emerging, hard-to-treat form of disseminated-CL [34]. In the Old World, several cases are literary reported as “disseminated-CL,” but those are co-infection with HIV, caused by *L.* (*L.*) *major* or other unknown species of the subgenus *Leishmania* [35]. The cases are different from those found in the New World.

In the current study, rK39 rapid kit test revealed positive bands both for the sera of diffuse-CL and disseminated-CL patients. The kit test using *L.* (*L.*) *chagasi* antigen is prepared for the qualitative detection of antibodies to *L.* (*L.*) *donovani* complex in human serum (InBios International, Instructions). However, we applied the test to our cases, expecting the cross-reactions between the *Leishmania* species, and observed strong positive bands in the cases. Furthermore, in our preliminary study, positive reaction of rK39 kit test against the serum of a MCL patient was also observed, but no positive reaction was found in the sera of localized-CL and mucosal (ML) patients; both are acute and/or relatively mild cases of CL (Hashiguchi et al., unpublished data). Cross-reactions of the rK39 kit test are reported at some extent. For example, positivity in at least one test employing recombinant (rK39) antigen was observed in 24 (45 %) patients with CL from Venezuela and Brazil and 47 (82.4 %) with *Plasmodium vivax* or *P. falciparum* from Brazil [36]. On the contrary, using the rK39 kit test, 100 % of the samples from 272 subjects with confirmed CL was negative, confirming the absolute absence of a serological cross-reaction [37]. Based on these results, Molinet et al. proposed the recommendation that the lack of a cross-reaction in CL patients infected by parasites of the same genus highlights the specificity of the rK39 antigen for the diagnosis of VL in areas with the sympatric circulation of *L.* (*V.*) *braziliensis* and *L.* (*L.*) *infantum* in Brazil [37]. Furthermore, Maia et al. mentioned that the rK39 proteins used either in a strip test or in an ELISA and the DAT are the best choices for implementation of rapid, easy, and efficient test for serodiagnosis of VL, performing a systematic review and meta-analysis of the studies published [38].

## Conclusions

In conclusion, the terms, diffuse-CL and disseminated-CL, should be used more correctly, based on the clinical, parasitological, and immunological features of the two forms. Furthermore, diffuse-CL is highly resistant to the drugs, and no specific and efficient medication is available for this disease form in the world. Therefore, the diffuse-CL should be diagnosed precisely at the early phase of the disease, differentiating from disseminated-CL which is sensitive against the available drugs.
